# Nurses' prediction of volume status after aneurysmal subarachnoid haemorrhage: a prospective cohort study

**DOI:** 10.1186/cc7142

**Published:** 2008-12-01

**Authors:** Reinier G Hoff, Gabriel JE Rinkel, Bon H Verweij, Ale Algra, Cor J Kalkman

**Affiliations:** 1 Department of Perioperative & Emergency Care, Rudolf Magnus Institute of Neuroscience, University Medical Center Utrecht, Heidelberglaan, Utrecht, 3584 CX, The Netherlands; 2 Department of Neurology, Rudolf Magnus Institute of Neuroscience, University Medical Center Utrecht, Heidelberglaan, Utrecht, 3584 CX, The Netherlands; 3 Department of Neurosurgery, Rudolf Magnus Institute of Neuroscience, University Medical Center Utrecht, Heidelberglaan, Utrecht, 3584 CX, The Netherlands; 4 Julius Center for Health Sciences and Primary Care, University Medical Center Utrecht, Heidelberglaan, Utrecht, 3584 CX, The Netherlands

## Abstract

**Introduction:**

Patients who have suffered aneurysmal subarachnoid haemorrhage (SAH) often have derangements in blood volume, contributing to poor outcome. To guide fluid management, regular assessments of volume status must be conducted. We studied the ability of nursing staff to predict hypovolaemia or hypervolaemia, based on their interpretation of available haemodynamic data.

**Methods:**

In a prospective cohort study, intensive care unit and medium care unit nurses, currently treating patients with recent SAH, were asked to predict present volume status. For their assessment they could use all available haemodynamic parameters (for example, heart rate, blood pressure, fluid balance). The nurses' assessments were compared with the actual circulating blood volume (CBV), as measured daily with pulse dye densitometry during the first 10 days after SAH. Normovolaemia was defined as a CBV of 60 to 80 ml/kg body weight; hypovolaemia as CBV under 60 ml/kg; severe hypovolaemia as CBV under 50 ml/kg and hypervolaemia as CBV above 80 ml/kg.

**Results:**

A total of 350 combinations of volume predictions and CBV measurements were obtained in 43 patients. Prediction of hypovolaemia had a sensitivity of 0.10 (95% confidence interval [CI] = 0.06 to 0.16) and a positive predictive value of 0.37 (95% CI = 0.23 to 0.53) for actual hypovolaemia. The prediction of hypervolaemia had a sensitivity of 0.06 (95% CI = 0.01 to 0.16) and a positive predictive value of 0.06 (95% CI = 0.02 to 0.19) for actual hypervolaemia. Mean CBV was significantly lower in instances considered hypervolaemic than in instances considered normovolaemic.

**Conclusions:**

Assessment of haemodynamic condition in patients with SAH by intensive care unit or medium care unit nurses does not adequately predict hypovolaemia or hypervolaemia, as measured using pulse dye densitometry. Fluid therapy after SAH may require guidance with more advanced techniques than interpretation of usual haemodynamic parameters.

## Introduction

Patients with aneurysmal subarachnoid haemorrhage (SAH) often have derangements in blood volume [1]. Hypovolaemia in these patients is associated with a greater risk for delayed cerebral ischaemia, whereas hypervolaemia increases the risk for pulmonary oedema and cardiac failure [2]. Fluid management after SAH is therefore aimed at maintaining normovolaemia [3]. To guide fluid management, a regular and accurate assessment of current volume status must be conducted, and such assessments are usually based on the available haemodynamic data. In our experience, nurses are often involved in these assessments and in decisions on fluid management. We studied the ability of nursing staff to predict hypovolaemia or hypervolaemia adequately in patients with SAH.

## Materials and methods

We conducted a prospective cohort study in patients admitted within 72 hours after aneurysmal SAH. The study setting was the 30-bed general intensive care unit (ICU; 150 nurses) and the seven-bed neurological medium care unit (MCU; 18 nurses) of the University Medical Center Utrecht. The hospital has a case load of around 150 SAH patients per year. Patients with SAH in good or reasonable clinical condition (World Federation of Neurological Surgeons grades 1 to 3) were mostly admitted to the MCU; patients in poorer condition (World Federation of Neurological Surgeons grade 4 or 5) were admitted to the ICU, as were patients in need of artificial ventilation or inotropic support.

The Medical Ethics Research Committee of the University Medical Center Utrecht approved the study. Written informed consent was obtained from the patients or, in case of impaired consciousness, from legal representatives. The study period was from days 1 to 10 after the SAH. Patients were treated according to current standard therapy, aimed at early treatment of the aneurysm by coiling or clipping and maintenance of normal vital functions. The goal of fluid management was to maintain normovolaemia. Fluid administration was adjusted on the basis of fluid balance, calculated at 6-hour intervals, by subtracting urinary volume from total oral and intravenous intake. The aim was to keep the daily fluid balance at 750 ml positive, in order to compensate for insensible fluid loss through perspiration and respiration. When the patient developed a fever (for >6 hours), the desired level for daily fluid balance was increased by 500 ml for each degree Celsius above 37°C to allow for increased insensible loss.

Nurses could participate in the study if they had finished their supplementary training as ICU or MCU nurse. They were asked to complete a brief questionnaire, indicating their opinion on current volume status as hypovolaemic, normovolaemic or hypervolaemic. Nurses were allowed to use all available parameters to form their opinion but they were asked to refrain from consulting other nurses or doctors. Parameters the nurses used included heart rate, arterial and central venous blood pressures, fluid balance, urine production and the presence of oedema. On each day during the study period, only one questionnaire could be completed by each individual nurse for the one patient who this nurse was taking care of during that day. The questionnaire was linked to the patient but no data on individual nurses were collected, to ensure anonymity of the nurses and thereby removing any fear that data could be used for individual quality control. The nurses were not informed about the accuracy of their predictions.

Circulating blood volume (CBV) was measured daily using pulse dye densitometry, a bedside dye dilution technique that has previously been validated and used in patients after SAH [4-6]. Normovolaemia was defined as a measured CBV of 60 to 80 ml/kg body weight, hypovolaemia as CBV under 60 ml/kg, severe hypovolaemia as CBV under 50 ml/kg and hypervolaemia as CBV above 80 ml/kg [7-9].

We compared the nurses' predictions of volume status with the actual CBV. We considered the combinations of the nurses' predictions with the measured CBV values (denoted hereafter on as 'instances') to be independent observations, because different nurses assessed volume status on different days.

For analysis, we compared mean CBV between instances that were considered hypovolaemic, normovolaemic or hypervolaemic, and we calculated mean differences with corresponding 95% confidence intervals (CIs), taking normovolaemia as the reference. We calculated the prior probability, sensitivity, specificity, positive and negative predictive values (with their corresponding 95% CIs) for the prediction of hypovolaemia or hypervolaemia. Prior probability was defined as the number of instances with the condition (hypovolaemia or hypervolaemia) present, as a proportion of the total number of instances. Sensitivity was the probability that the prediction was positive (hypovolaemia or hypervolaemia present) if the predicted condition was actually present. Specificity was the probability that the prediction was negative (no hypovolaemia or no hypervolaemia) if the condition was absent. Positive predictive value was the probability for any particular positive prediction (hypovolaemia or hypervolaemia present) that it was correct (true positive). Negative predictive value was the probability for any particular negative prediction (no hypovolaemia or no hypervolaemia) that the condition was indeed absent (true negative).

Calculations were made using VassarStats: Website for Statistical Computations [10]. These calculations were made for all instances combined, and separately for instances in the absence or presence of artificial ventilation or inotropics.

## Results

Between January 2006 and June 2007, nurses' questionnaires were collected for 43 patients. Clinical characteristics are provided in Table 1. The study period of 10 days was completed by 38 patients (88%); three patients died within the study period, one patient withdrew consent and one patient was transferred to another hospital.

**Table 1:** Patient characteristics

Parameter/characteristic	Value
Number of patients	43
Women (*n *[%])	32 (74%)
Age (years; mean ± SD)	56.6 ± 14.0
Clinical condition on admission (*n *[%])	
WFNS-1	22 (51%)
WFNS-2	6 (14%)
WFNS-3	4 (9%)
WFNS-4	9 (21%)
WFNS-5	2 (5%)
Treatment of the aneurysm (*n *[%])	
Coiling	27 (63%)
Clipping	13 (30%)
Outcome at 3 months after SAH (*n *[%])	
mRS-0	2 (5%)
mRS-1	10 (23%)
mRS-2	8 (19%)
mRS-3	10 (23%)
mRS-4	0 (0%)
mRS-5	5 (12%)
Dead	8 (19%)

mRS, modified Rankin Scale; SD, standard deviation; WFNS, World Federation of Neurological Surgeons grading scale.

In all, 350 combinations of a completed questionnaire and a CBV measurement were obtained. CBV varied considerably in individual patients. None of the 43 included patients had all measurements within the normovolaemic range (60 to 80 ml/kg). Twelve patients (28%) had blood volume measurements during the study period that were spread over the hypovolaemic, normovolaemic and hypervolaemic ranges. Fifteen patients (35%) had measurements in both the hypovolaemic and normovolaemic range; nine patients (21%) had measurements in both the normovolaemic and hypervolaemic range; and seven patients (16%) only had measurements indicating hypovolaemia. Also, the predictions by nurses of volume status varied considerably on consecutive days. In only nine patients (21%) was normovolaemia considered to be present by the nurses on all measurement days.

Table 2 presents a comparison of mean CBV for the instances classified by the nurses as hypovolaemic, normovolaemic or hypervolaemic. If nurses predicted hypervolaemia, then the mean CBV was 8.4 ml/kg (95% CI = 3.7 ml/kg to 13.1 ml/kg) lower than if they predicted normovolaemia. There was no significant difference in mean CBV between hypovolaemic and normovolaemic predictions.

**Table 2:** Predicted volume status and measured CBV

Predicted volume status	Predictions (*n *[%])	CBV (ml/kg; mean ± SD)
Hypovolaemia	41 (12%)	66.9 ± 16.9
Normovolaemia	262 (75%)	65.0 ± 15.2
Hypervolaemia	47 (13%)	56.6 ± 14.3

CBV, circulating blood volume; SD, standard deviation.

Table 3 presents the test characteristics for the nurses' predictions of hypovolaemia or hypervolaemia. Of 41 hypovolaemic predictions, measured CBV was in 15 instances within the hypovolaemic range (CBV <60 ml/kg) and was in six instances within the severe hypovolaemic range (CBV <50 ml/kg). Of the 309 instances with predicted normovolaemia or hypervolaemia, 139 had measured hypovolaemia, and 57 of these instances were severe hypovolaemia. Of the 47 hypervolaemic predictions, hypervolaemia was measured in three. Of the 303 predictions of normovolaemia or hypovolaemia, measured CBV was within the hypervolaemic range in 51 instances.

**Table 3:** Predictive values

Predicted and measured CBV values	Value (95% CI)
Predicted hypovolaemia and measured hypovolaemia (CBV <60 ml/kg)	
Prior probability	0.44 (0.39 to 0.49)
Sensitivity	0.10 (0.06 to 0.16)
Specificity	0.87 (0.81 to 0.91)
Positive predictive value	0.37 (0.23 to 0.53)
Negative predictive value	0.55 (0.49 to 0.61)
Predicted hypovolaemia and measured severe hypovolaemia (CBV <50 ml/kg)	
Prior probability	0.18 (0.14 to 0.23)
Sensitivity	0.10 (0.04 to 0.20)
Specificity	0.88 (0.83 to 0.91)
Positive predictive value	0.15 (0.06 to 0.30)
Negative predictive value	0.82 (0.77 to 0.86)
Predicted hypervolaemia and measured hypervolaemia (CBV >80 ml/kg)	
Prior probability	0.15 (0.12 to 0.20)
Sensitivity	0.06 (0.01 to 0.16)
Specificity	0.85 (0.80 to 0.89)
Positive predictive value	0.06 (0.02 to 0.19)
Negative predictive value	0.83 (0.78 to 0.87)

CBV, circulating blood volume.

In 47 instances (13%) artificial ventilation was used and in 32 instances (9%) inotropics. For instances with or without artificial ventilation, and with or without inotropics, there were essentially no differences in sensitivity, specificity or predictive values for the nurses' predictions.

## Discussion

The interpretation of volume status by ICU or MCU nurses does not correspond with the actual presence of hypovolaemia or hypervolaemia in patients with SAH. Deviations from normovolaemia occurred frequently, but most instances were not recognized as such, which resulted in a very low sensitivity of prediction. The positive predictive values of the nurses' predictions were even slightly lower than the prior probabilities of (severe) hypovolaemia or hypervolaemia. If hypervolaemia was predicted, then in fact a statistically significant lower CBV was found than if normovolaemia or hypovolaemia was predicted. In most instances no (severe) hypovolaemia or hypervolaemia was present. Therefore, a negative prediction (no hypovolaemia or no hypervolaemia) was usually correct, resulting in higher values for specificity and higher negative predictive values.

Assessment of the patient's condition is a fundamental part of critical care nursing, and optimizing haemodynamic status should be viewed as a team effort [11]. One of the important factors determining quality of the circulation is the amount of circulating blood [8]. We defined normovolaemia as a measured CBV of 60 to 80 ml/kg body weight, in accordance with the findings of previous studies in which a value of approximately 70 ml/kg for adults was identified [5,7-9]. This definition of 'normal blood volume' is a simplification because blood volume varies depending on age, sex and build. Furthermore, the changes in blood volume that occur in critical illness are incompletely understood [12]. Therefore, we used fairly wide margins (60 to 80 ml/kg) in our definition of normovolaemia, and we defined the threshold for severe hypovolaemia (<50 ml/kg) in accordance with the level that was previously shown to be associated with an increased risk for secondary ischaemia after SAH [13].

None of the clinical signs normally used to monitor the circulation (for example, arterial or venous pressure) exhibits a consistent relation with fluid responsiveness or with measured blood volume [14]. Dynamic indicators such as pulse pressure variation may have a better relation with fluid responsiveness in critically ill patients, but the relation with blood volume is not yet clear [15]. Blood volume itself, albeit an important determinant of preload, is only one of the factors that determines the adequacy of tissue perfusion. To evaluate current volume status, many haemodynamic parameters must be taken into consideration together and interpreted within the context of the patient's overall clinical condition [16]. This interpretation therefore remains quite difficult, as is underscored by our findings.

A limitation of our study is that the 350 combinations of CBV measurements and nurses' predictions were obtained from 43 patients. In each patient multiple CBV measurements were made, albeit on different days, and therefore these are not independent measurements in a strict sense. However, for practical purposes we considered the combinations of these daily measurements with the nurses' predictions to be independent observations because of the large variation in measured blood volume in individual patients on consecutive days, the large number of nurses who made the predictions and the large variation in the predictions that were made.

We did not collect data on nurses' motivations for predicting hypovolaemia or hypervolaemia. Most nurses have ample experience with this patient category because our hospital has a relatively large annual load of patients who have suffered SAH. We cannot explain with any certainty the large discrepancy between prediction and measured CBV. An explanation might be that because patients were managed in accordance with a fluid policy based on fluid balances, a more positive fluid balance may have been seen as an indication for hypervolaemia. In a previous study of CBV after SAH, the relation between fluid balance and CBV was actually very poor [13]. Furthermore, we cannot ascertain whether the observed low predictive values are the result of a poor correlation between haemodynamic parameters available to the nurses and measured blood volume, or of poor interpretation of these parameters by the nurses. We did not study whether the treating physicians were more accurate in their predictions.

## Conclusion

Hypovolaemia and hypervolaemia occurred frequently after SAH but were often not recognized as such. The nurses' predictions of current volume status do not seem sufficiently reliable to serve as a basis for therapeutic decisions. More advanced techniques for bedside assessment of volume status may be indicated for optimizing volume status in patients with SAH.

## Key messages

• Both hypovolaemia and hypervolaemia occur frequently in patients after recent SAH.

• Qualified ICU and MCU nurses, interpreting conventional haemodynamic parameters to estimate volume status, are not able to recognize the presence of hypovolaemia or hypervolaemia reliably.

• The interpretation of current volume status by the nurses can only play a limited role in the guidance of fluid policy.

## Abbreviations

CBV: circulating blood volume; CI: confidence interval; ICU: intensive care unit; MCU: medium care unit; SAH: aneurysmal subarachnoid haemorrhage.

## Competing interests

The authors declare that they have no competing interests.

## Authors' contributions

All of the authors were involved in designing the study. RH collected the data and drafted the manuscript. AA was involved in statistical analysis. All authors were involved in interpretation of the data. GR, BV, AA and CK revised the manuscript. All authors approved the final manuscript.
